# Evaluating the Relationship Between Fitbit Sleep Data and Self-Reported Mood, Sleep, and Environmental Contextual Factors in Healthy Adults: Pilot Observational Cohort Study

**DOI:** 10.2196/18086

**Published:** 2020-09-29

**Authors:** Darshan Thota

**Affiliations:** 1 Madigan Army Medical Center Joint Base Lewis-McChord, WA United States

**Keywords:** Fitbit, sleep, healthy, mood, context, waking

## Abstract

**Background:**

Mental health disorders can disrupt a person’s sleep, resulting in lower quality of life. Early identification and referral to mental health services are critical for active duty service members returning from forward-deployed missions. Although technologies like wearable computing devices have the potential to help address this problem, research on the role of technologies like Fitbit in mental health services is in its infancy.

**Objective:**

If Fitbit proves to be an appropriate clinical tool in a military setting, it could provide potential cost savings, improve clinician access to patient data, and create real-time treatment options for the greater active duty service member population. The purpose of this study was to determine if the Fitbit device can be used to identify indicators of mental health disorders by measuring the relationship between Fitbit sleep data, self-reported mood, and environmental contextual factors that may disrupt sleep.

**Methods:**

This observational cohort study was conducted at the Madigan Army Medical Center. The study included 17 healthy adults who wore a Fitbit Flex for 2 weeks and completed a daily self-reported mood and sleep log. Daily Fitbit data were obtained for each participant. Contextual factors were collected with interim and postintervention surveys. This study had 3 specific aims: (1) Determine the correlation between daily Fitbit sleep data and daily self-reported sleep, (2) Determine the correlation between number of waking events and self-reported mood, and (3) Explore the qualitative relationships between Fitbit waking events and self-reported contextual factors for sleep.

**Results:**

There was no significant difference in the scores for the pre-intevention Pittsburg Sleep Quality Index (PSQI; mean 5.88 points, SD 3.71 points) and postintervention PSQI (mean 5.33 points, SD 2.83 points). The Wilcoxon signed-ranks test showed that the difference between the pre-intervention PSQI and postintervention PSQI survey data was not statistically significant (Z=0.751, *P*=.05). The Spearman correlation between Fitbit sleep time and self-reported sleep time was moderate (r=0.643, *P*=.005). The Spearman correlation between number of waking events and self-reported mood was weak (r=0.354, *P*=.163). Top contextual factors disrupting sleep were “pain,” “noises,” and “worries.” A subanalysis of participants reporting “worries” found evidence of potential stress resilience and outliers in waking events.

**Conclusions:**

Findings contribute valuable evidence on the strength of the Fitbit Flex device as a proxy that is consistent with self-reported sleep data. Mood data alone do not predict number of waking events. Mood and Fitbit data combined with further screening tools may be able to identify markers of underlying mental health disease.

## Introduction

Anxiety, depression, and post-traumatic stress disorder (PTSD) are increasingly common types of mental health disorders in the United States. Mental health disorders can cause significant reduction in function and quality of life [1].While mental health disease is prevalent throughout the United States, it affects a disproportionately large proportion of the active duty service member (ADSM) population. In 2013, 20% of ADSM had a mental health disease, with the Army showing the highest prevalence of mental health disease, at 30% [2]. Routine screening and early referral for mental health disorders can help improve quality of life for ADSM [3]. Since it is known that treatment is effective, the key to better serving ADSM is early identification of underlying mental health disorders.

There is a well-defined relationship between mental health disorders, such as anxiety, depression, and PTSD, and sleep disturbances [4]. Up to 90% of patients with major depressive disorder have sleep disturbances [5]. Patients with generalized anxiety disorder have double the risk of having sleep disturbances as compared to healthy controls [6]. A majority of patients with PTSD suffer from sleep disturbances [7]. Sleep disturbances due to underlying mental health conditions can be measured using contextual factors such as fear, worry, or life stressors.

Personal fitness devices are wearable sensors (usually worn on the wrist) that allow for direct tracking of biometric data. Devices such as the Fitbit are light, portable, and inexpensive and can obtain passive sleep data [8]. These trackers have been used by both the Army and Navy to promote health and are ubiquitous in the ADSM population [9]. Specifically, Fitbit has been found to be a valid method of obtaining sleep metrics compared to the gold standard polysomnography (PSG) [10-12]. Fitbits have also been found to positively correlate with patient-reported outcomes such as sleep and step count [13]. Fitbit has an intradevice reliability of 96.5%-99.1% for measuring sleep efficiency and total sleep time compared with the gold standard of PSG in a study of 24 healthy volunteers [10]. Another study compared the Fitbit Flex, the same device used in this study, against PSG and found a correlation of 97.4% for total sleep time in good sleepers and 88.6% for participants with insomnia [14].

Many studies have measured total sleep time, time awake, and number of waking events but have not investigated causes for why the waking events happened [15]. A study of 22 participants using the SleepTight tracking algorithm to evaluate contextual factors such as caffeine and exercise did not show a statistically significant correlation with good, neutral, or poor sleep over a month [16]. Another study also attempted to explore the relationship between sleep and contextual factors with 12 participants looking at Fitbit data over 2 weeks compared with an online sleep tool called Sleep Explorer [15]. The most common reasons for increased number of waking events were spouses, children, pets, garbage trucks, and alcohol [15]. An additional study by the same author found that the total time slept and number of waking events most strongly correlated with the participants’ experience of sleep [17]. Finally, a small study of 10 participants looking at Fitbit Alta sleep metrics and self-reported sleep diaries found a positive correlation between the 2 measures [18]. None of these studies attempted to explore the relationship between self-reported mood, sleep, and contextual factors as markers of early mental health disorders.

Although Fitbit has been found to be a valid instrument for measuring sleep data, it has not been used to evaluate potential underlying mental health disorders. The paucity of data creates an opportunity to design research studies to fill current gaps in the literature.

This study aimed to evaluate if Fitbit sleep data can be validated as reliable compared with self-reported sleep and mood. If Fitbit can be found to be a reliable device that can uncover early markers for mental health disorders, then Fitbit could be used as a screening tool to identify early markers of mental stress in different populations such as ADSM. No analysis in the literature has attempted to address the following 3 aims: (1) Determine the correlation between daily Fitbit minutes slept and daily self-reported sleep data, (2) Determine the correlation between Fitbit number of waking events and self-reported mood, and (3) Explore qualitative relationships between Fitbit waking events and self-reported contextual factors for sleep.

## Methods

This observational cohort study evaluated the relationship among Fitbit sleep data (hours and waking events), self-reported mood, self-reported sleep (hours and waking events), and self-reported environmental contextual factors that impact sleep in a convenience sample of healthy, working, adult ADSM participants over a 2-week period at the Madigan Army Medical Center. Healthy participants were defined as not having primary uncontrolled insomnia, obstructive sleep apnea, hyperthyroidism, or cardiac dysrhythmia. Inclusion criteria consisted of voluntary participation in the study and having a functioning Fitbit. Exclusion criteria included having a pre-existing uncontrolled primary sleep disorder, uncontrolled obstructive sleep apnea, uncontrolled thyroid disease, dysrhythmias, or nonfunctioning Fitbit. Exclusion criteria were based on studies with a similar design [15].

Participants wore the Fitbit Flex for the 2-week data collection period. Data were collected from participants at 3 time points: pre-intervention, during the intervention, and postintervention.

Participants completed pre-intervention and postintervention sleep surveys using the Pittsburg Sleep Quality Index (PSQI) [19]. Participants completed daily mood and sleep logs using the National Institute of Mental Health’s Life Chart Method (NIMH LCM) [20]. At the end of the first week of data collection and at the end of the 2-week period, participants completed the Clinical Interview Schedule-Revised (CIS-R) [21] as a measure of contextual factors for sleep.

### Aim 1. Determine the Correlation Between Daily Fitbit Sleep Data and Daily Self-Reported Sleep

The relationship between daily Fitbit minutes slept and daily self-reported number of hours slept was assessed using Spearman correlation analysis. Spearman correlation analysis was used to compare ordinal self-reported sleep to measured Fitbit sleep. As a quality check, the pre-intervention PSQI and postintervention PSQI scores were compared using the Wilcoxon signed rank test to ensure no change in pre-intervention and postintervention sleep habits during the 2-week interval. The Wilcoxon signed ranked test was used to compare ordinal values from the pre-intervention and postintervention PSQI surveys.

### Aim 2. Determine the Correlation Between Number of Fitbit Waking Events and Self-Reported Mood

The NIMH LCM was used to track self-reported mood with a daily log of the number of hours slept and a presleep mood assessment on a rating scale from –3 (lowest) to +3 (highest). The relationship between daily Fitbit number of waking events and daily self-reported presleep mood were assessed with the Spearman correlation.

### Aim 3. Explore Qualitative Relationships Between Fitbit Waking Events and Self-Reported Contextual Factors for Sleep

Contextual factors contributing to disruptive sleep were obtained using survey data from the PSQI and CIS-R. Both surveys allow for participants to pick from a list of common sleep disturbances as well as manually write in other contextual factors. These open-ended responses were analyzed and sorted into groups based on similarity to identify specific contextual factors that were disruptive to participant sleep.

## Results

A total of 17 participants (P1-P17) completed the study (Table 1). No self-reported data were excluded.

**Table 1:** Participant demographic profile (n=17).

Age bracket (years)	Number of participants	Number of men	Number of women
31-35	1	1	0
36-40	2	0	2
41-45	4	3	1
46-50	3	2	1
51-55	3	3	0
56-60	4	4	0

As seen in Table 1, the demographic analysis shows that participants were in the working age range, and the sample was approximately three-fourths male and one-fourth female. No significant demographic differences were seen.

Table 2 summarizes the survey and Fitbit sleep data. Disrupted sleep habits were reported by 5 participants (P2, P5, P11, P13, and P16), with both pre-intervention and postintervention PSQI sleep scores >5. These scores could be a sign of possible underlying conditions giving rise to disrupted sleep patterns. Contextual factors reported on the CIS-R by those participants indicate situational factors that contributed to disruptive sleep. Each participant with high pre-intervention and postintervention PSQI sleep scores indicating poor sleep reported situational confounding factors in the CIS-R survey (Table 2).

There was no significant difference in the mean scores for the pre-intervention PSQI (5.88, SD 3.71) and postintervention PSQI (5.33, SD 2.83). The Wilcoxon signed-ranks test showed that the difference between the pre-intervention PSQI and postintervention PSQI survey data was not statistically significant (Z=0.751, *P*=.05). Thus, participants’ self-reported sleep habits remained stable during the study period.

**Table 2:** Fitbit-reported data versus participant self-reported survey data.

Variables		Results
Fitbit hours slept, mean (SD)		6.50 (0.95)
Self-reported hours slept, mean (SD)		6.38 (0.80)
Fitbit waking events, mean (SD)		1.79 (1.05)
Self-reported mood (–3 to +3), mean (SD)		0.67 (1.01)
Pre-intervention PSQI^a^, mean (SD)		5.53 (2.83)
Postintervention PSQI, mean (SD)		5.88 (3.71)
**Interim CIS-R^b^ contextual factors, n**		
	Noises	2
	Pain	2
	Worries	4
	Other	9
**Postintervention CIS-R contextual factors, n**		
	Noises	3
	Pain	3
	Shift work/late nights	3
	Worries	2
	Other	6

^a^PSQI: Pittsburg Sleep Quality Index.

^b^CIS-R: Clinical Interview Schedule-Revised.

### Aim 1: Comparison of Self-Reported Sleep Versus Fitbit Sleep

**Figure 1:**
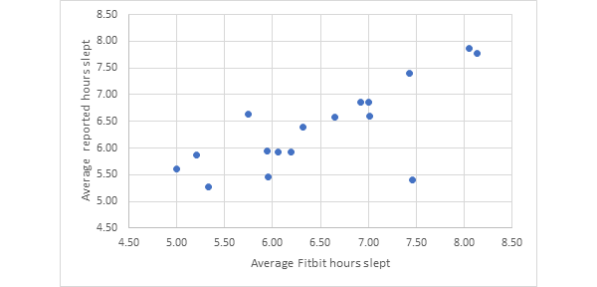
Average sleep recorded by Fitbit compared with average self-reported sleep (n=17).

Figure 1 compares Fitbit sleep time and self-reported sleep time. The population mean of Fitbit average hours slept was 6.49 hours (SD 0.95 hours), which was close to the participant self-reported mean of average hours slept of 6.38 hours (SD 0.80 hours). The Spearman correlation between Fitbit sleep time and self-reported sleep time was moderate (r=0.643, *P*=.005) [22].

### Aim 2: Comparison of Self-Reported Mood Versus Fitbit Waking Events

**Figure 2:**
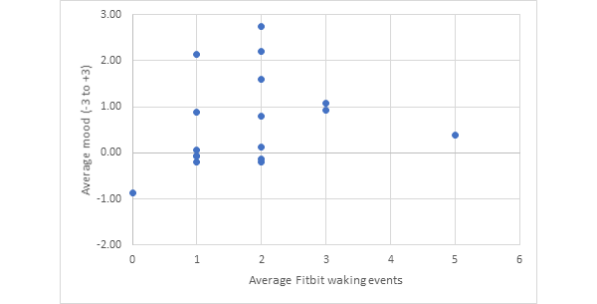
Average number of waking events compared with average self-reported mood (n=17).

The average number of Fitbit waking events was 1.79 (SD 1.05). The average self-reported mood was 0.67 (SD 1.00). Figure 2 shows no clear linear relationship between self-reported mood and number of waking events. This lack of relationship between waking events and self-reported mood is confirmed by the weak correlation between number of waking events and self-reported mood (r=0.354, *P*=.163).

### Aim 3: Exploring the Relationship Between the Number of Waking Events and Contextual Factors

Qualitative analysis of the interim and post CIS-R data from Table 2 resulted in 4 categories of contextual factors: noises, pain, shift work/late nights, and worries. P9, P12, and P13 reported “noises” were the main reason to wake up at night. “Noises” and “shift work/late nights” provide additional environmental context for waking events, while “pain” provides an additional physical context for waking events. The subcategories of “worries” provide additional mental health context for waking events.

**Figure 3:**
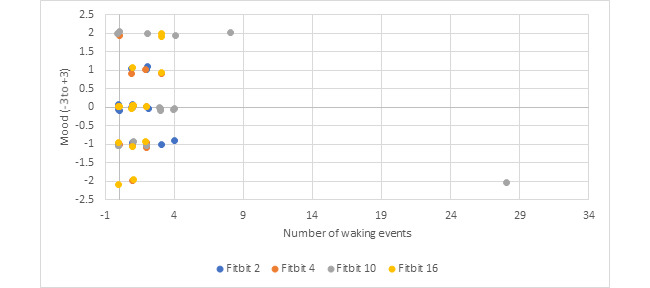
Mood compared with number of waking events for the subcategory of the contextual factor "worries".

A subset of participants was evaluated to further explore “worries” as a mental health marker of stress. The correlations between the number of waking events and mood were analyzed for this subset of participants and are shown in Figure 3.

Figure 3 shows the relationship between number of waking events and presleep mood for the subcategories of “worries.” The mental resilience of this population is seen in the relationship between high mood and an increased number of waking events. The outlier may indicate a potential marker of mental stress and possibly an underlying mental health disorder.

## Discussion

There was no statistical difference in the pre-intervention PSQI and postintervention PSQI scores. This indicates that sleep habits were stable, even with the use of a Fitbit. Additionally, Fitbit hours slept correlated well with self-reported hours slept. This finding for Aim 1 indicates that the Fitbit Flex functioned effectively as a feasible tool for measuring participants’ self-reported sleep and could be reliably used for further testing in future populations. The unique contribution of Aim 1 is that it showed a correlation between self-reported sleep and measured sleep in a population of healthy, working-age individuals at the Madigan Army Medical Center. Previous literature has shown a positive correlation between self-reported sleep and mood in the general population suing PSG and actigraphy. However, no study has aimed to confirm the correlation between self-reported sleep and mood in a healthy, working age population in the military health system. Confirming that this correlation exists provides the foundation for trusting Fitbit data as a reliable tool for measuring sleep and serves as a baseline to explore other parameters such as number of waking events and presleep mood.

Aim 2 attempted to determine whether mood can be used as a marker for detecting underlying mental health disorders. The idea being that if someone has anxiety, depression, PTSD, or another mental health disorder, this will be observed as a negative mood prior to going to bed, and the Fitbit will be able to capture this negative mood as an increased number of waking events. However, although there was a moderate correlation for Aim 1, as measured using the Spearman coefficient, the correlation for Aim 2 was weak.

This finding of a weak correlation between number of waking events and presleep mood indicates that self-reported mood does not correlate with increased waking events in this population. This indicates that presleep mood is not a reliable marker for detecting early markers of mental health disorders. However, given the lack of variability in the population data, this cohort may be used a potential baseline for future testing.

Additionally, 2 unintended findings occurred while investigating Aim 2. Since this was a healthy population without underlying mental health disorders and no significant mood or waking event relationship was found, this population can be used as a baseline for measuring normal population data. This can be helpful for future studies looking at different mood, sleep, and contextual factors.

Interestingly, several participants reported a higher mood with an increased number of waking events. One potential explanation of this is the sample population itself. The study participants were healthy adults serving in an Armed Forces environment. This cohort may have had naturally positive attitudes, becoming energized during times of stress. For this group, waking events do not increase with a negative presleep mood possibly due to the mental resilience and positive stress response of this population under stressful conditions. This study’s findings could help to screen populations for stress-resilient personnel by further testing using the Fitbit device, mood, and contextual factors in similar populations. These findings may also be of significance for use as baseline in healthy, working-age, military-associated populations. Further testing with the ADSM population could attempt to screen for less adaptive stress responses that show a correlation between negative mood and increased waking events.

Finally, for Aim 3, the contextual factors disrupting sleep varied by participant and lifestyle context. Specific common disturbances identified were “noises,” “pain,” “shift work/late nights,” and “worries.” “Pain” and “worries” may indicate underlying physical and mental stress. A subanalysis performed with data from these participants found outliers in the number of waking events. Although the aggregate population data did not show a correlation between mood and waking events, at the individual level, a potential correlation may exist as identified by P10’s increased number of waking events and self-report of “worries.” Future studies may use an additional dedicated mental health screening tool such as the Patient Health Questionnaire (PHQ-9), which may reveal a relationship between mood and waking events that Aim 2 did not show.

### Limitations

There are several limitations to this study. First, the sample size was small, consisting of 17 participants. However, this sample size is consistent with similar studies [15]. Further testing with a larger sample size may be needed to generalize findings to the ADSM population.

Second, the NIMH LCM is used primarily to track the effectiveness of treatments in patients with bipolar disease. Here, it was used to track daily mood, although its intended purpose is to track the severity of bipolar over a period of a month. Since mood data were collected from self-reported surveys, recall bias may have affected the accuracy of these results. Future studies may consider obtaining longitudinal mood data rather than a single mood value.

Third, both the PSQI and CIS-R contain questions asking participants to describe contextual factors that may disrupt sleep over a 30-day period. Participants were asked to report contextual factors for 2 weeks only. Since portions of the PSQI and CIS-R were adapted for a shorter study period, this may limit their validity.

Fourth, since this study first focused on healthy participants prior to being conducted in patients with known pathology, only a few mood scores were found in the –1 to –3 region. This may limit the ability to find a linear relationship in an unobserved area. Future studies may address this by evaluating patients with known underlying mental health disease.

Finally, the Fitbit Flex is one of the oldest Fitbit models. A newer Fitbit model may correlate better with presleep mood. Newer models may include more sensitive methods for detecting REM sleep and may also be more sensitive in determining the number of waking events.

Despite these limitations, this study has a number of strengths. The Fitbit was shown to be a reliable device for measuring sleep. Fitbit waking data and presleep mood data serve as a baseline for future studies. Finally, the combination of Fitbit data and survey data can identify outliers in subgroups that can be evaluated for mental health disorders with further screening tools. Further screening in this subgroup of participants with an additional dedicated mental health survey such as the PHQ-9 may be able to reveal underlying mental health disorders.

### Conclusions

Since both mental health disorders and Fitbits are prevalent in the ADSM population, personal fitness trackers may be able to capture patient-generated data for clinically meaningful outcomes. The purpose of this study was to evaluate the relationship between Fitbit sleep data, self-reported mood, and contextual factors that may disrupt sleep to determine if the Fitbit device can be used to identify early markers of mental health disorders. The findings contribute valuable evidence on the strength of the Fitbit Flex as a proxy that is consistent with self-reported sleep data. Low mood was not found to correlate with an increased number of waking events. Increased mood was observed when there was an increased number of waking events, indicating that this population may exhibit a stress resilience not seen in the general population, which future work should investigate. Additionally, the subgroup analysis indicated that this population can be used as a baseline to identify outliers in the characteristics of a healthy Department of Defense population’s sleep and mood patterns. Finally, using the Fitbit in combination with mood and contextual surveys may identify outliers that can be further screened with a dedicated mental health screening tool such as the PHQ-9.

## Abbreviations

ADSM: active duty service member
CIS-R: Clinical Interview Schedule-Revised
NIMH LCM: National Institute of Mental Health’s Life Chart Method
PHQ-9: Patient Health Questionnaire
PSG: polysomnography
PSQI: Pittsburg Sleep Quality Index
PTSD: post-traumatic stress disorder
